# Screening of protein kinase inhibitors identifies PKC inhibitors as inhibitors of osteoclastic acid secretion and bone resorption

**DOI:** 10.1186/1471-2474-11-250

**Published:** 2010-10-26

**Authors:** Mette G Sørensen, Morten A Karsdal, Morten H Dziegiel, Jean A Boutin, Olivier Nosjean, Kim Henriksen

**Affiliations:** 1 Nordic Bioscience A/S, Herlev Hovedgade 207, DK-2730 Herlev, Denmark; 2 Blodbanken, Copenhagen University Hospital, Blegdamsvej 9, DK-2100 Copenhagen, Denmark; 3 Institut de Recherche Servier (IdRS), 125 Chemin de Ronde, 78290 Croissy-sur-Seine, France

## Abstract

**Background:**

Bone resorption is initiated by osteoclastic acidification of the resorption lacunae. This process is mediated by secretion of protons through the V-ATPase and chloride through the chloride antiporter ClC-7. To shed light on the intracellular signalling controlling extracellular acidification, we screened a protein kinase inhibitor library in human osteoclasts.

**Methods:**

Human osteoclasts were generated from CD14+ monocytes. The effect of different kinase inhibitors on lysosomal acidification in human osteoclasts was investigated using acridine orange for different incubation times (45 minutes, 4 and 24 hours). The inhibitors were tested in an acid influx assay using microsomes isolated from human osteoclasts. Bone resorption by human osteoclasts on bone slices was measured by calcium release. Cell viability was measured using AlamarBlue.

**Results:**

Of the 51 compounds investigated only few inhibitors were positive in both acidification and resorption assays. Rottlerin, GF109203X, Hypericin and Ro31-8220 inhibited acid influx in microsomes and bone resorption, while Sphingosine and Palmitoyl-DL-carnitine-Cl showed low levels of inhibition. Rottlerin inhibited lysosomal acidification in human osteoclasts potently.

**Conclusions:**

In conclusion, a group of inhibitors all indicated to inhibit PKC reduced acidification in human osteoclasts, and thereby bone resorption, indicating that acid secretion by osteoclasts may be specifically regulated by PKC in osteoclasts.

## Background

Bone is continuously remodeled throughout life to react to stress on the skeleton and to repair microfractures [1-3]. Bone is resorbed by the osteoclasts and new bone is formed by the osteoblasts [4]. Bone resorption is mediated through acidification of the resorption lacunae by the osteoclasts. The mineralized bone matrix is dissolved by secretion of protons through a V-ATPase [5-8], which is followed by chloride transport through ClC-7 to maintain electroneutrality [9-13]. At the low pH in the resorption lacuna cathepsin K degrades the organic phase of the bone [14,15]. The importance of the acidification process in osteoclasts is illustrated by mutations in the a3 subunit of the V-ATPase and in ClC-7, which lead to osteopetrosis [12,13,16-18]. Furthermore, inhibitors of acid secretion by the osteoclasts have been shown to have promising effects, and are being investigated as potential drug candidates for osteoporosis at the moment [19,20].

The intracellular mechanism underlying acid secretion appears to involve Protein Kinase A (PKA) and Protein Kinase C (PKC) [21,22], as a study implicated PKA as a negative regulator of acid secretion in rat osteoclasts [23], and another study showed effects with different tyrosine kinase inhibitors in avian osteoclasts [24]. PKC has also been implicated in the acid secretion process in avian osteoclasts, an effect related to reduction of V-ATPase activity [25]. In avian osteoclasts the tyrosine kinase c-src regulates osteoclastic acid secretion through the chloride channel CLIC5b [26], however, these findings appear to be specific for the avian osteoclasts as they were not reproduced in a human osteoclast based system [27], where ClC-7 appears to be the chloride channel of importance [10,28]. In summary there is no consensus on the intracellular control of acid secretion in human osteoclasts.

We investigated whether protein kinases play roles in mature human osteoclasts, and whether the roles are related to acid secretion using inhibitors of these kinases and their specific isoform. We used a panel of protein kinase inhibitors in acridine orange based acid secretion assays in whole cells and membrane fractions, as well as human osteoclasts seeded on cortical bone slices to evaluate the effect of the inhibitors on bone resorption.

## Methods

### Chemicals

Chemicals were obtained from SIGMA-ALDRICH A/S and culture media from LIFE TECHNOLOGIES A/S unless specified. Bafilomycin was obtained from Tocris, while the different kinase inhibitors were obtained from BIOMOL International LP.

### Cell culture

The CD14+ isolation was performed as previously described [29]. Briefly, the monocytes were isolated from peripheral blood by centrifugation on a Ficoll-Paque gradient (Amersham Pharmacia), and magnetically sorted using a CD14+ magnetic bead isolation kit (Dynal Biotech). The cells were then seeded in 75 cm^2 ^flasks, and cultured in αMEM containing 10% fetal calf serum, 100 units/mL penicillin, 100 μg/mL streptomycin and 25 ng/ml of M-CSF for three days, then they were lifted using trypsin and a cell scraper, and cultured until day 10 in the presence of 25 ng/ml M-CSF and 25 ng/ml RANKL (R&D Systems) unless otherwise stated.

The blood was received from the blood bank at the University Hospital of Copenhagen from volunteer donors, which all sign informed consent that the blood can be used for research purposes. The approval is held by the University Hospital of Copenhagen.

### Osteoclast resorption

Mature human osteoclasts were lifted from culture flasks and subsequently seeded on cortical bovine bone slices at a density of 20,000 cells/cm^2 ^and then culture for 5 days, with refreshment of medium once. The supernatant was collected and the release of calcium was measured. Inhibitors of resorption were added in the medium at different concentrations and compared to vehicle treated osteoclasts (DMSO).

### Cortical bovine bone slices

The bone slices were cut from sticks (Nordic Bioscience A/S), which were made of the cortical bone from cows. The sticks were cut into small slices with a thickness of 0.2 mm with a diameter that fits into 96 well plates.

### Measurement of calcium

The concentration of total calcium was measured in the culture supernatants after resorption using a colorimetric assay and a Hitachi 912 Automatic Analyzer (Roche Diagnostics).

### Osteoclast acidification assay

Acridine orange (3,6-bis[Dimethylamine]acridine) at 10 μg/ml was loaded for 45 min in the culture medium in the presence or absence of various inhibitors as described previously [27]. The dye was washed away and pictures were taken using an Olympus IX-70 microscope and an Olympus U-MWB filter (x20 objective), or fluorescence was measured using the SpectraMax M5 (Molecular Devices) at excitation 492 nm and emission 535 nm. The results are presented as percentage of the signal obtained with the positive control Bafilomycin treated condition.

### AlamarBlue assay

To assess cell viability AlamarBlue measurements were performed according to the manufacturer's protocol (Trek Diagnostics Systems Inc.). Briefly, AlamarBlue was diluted 1 to 10 in the cell culture medium, and the color change was monitored carefully. When a switch from blue to purple was observed, the color changes were measured using a plate reader (excitation wavelength 540 nm, emission 590 nm). Medium without cells was used as background. The cell viability was measured in mature human osteoclasts seeded on bone slices after the 5-day culture period at termination.

### Osteoclast microsomes

The osteoclast-derived membrane vesicles were isolated using a modification of a protocol published by [28]. Briefly, the mature cells were washed two times in PBS, and the cells were lifted by scraping in 10 mM Tris-HCl, 4 mM EDTA pH7.4 containing Complete Mini EDTA-free protease inhibitor tablet. The collected cells were then homogenized using an Ultraturrax blender and a Teflon homogenizer, and then the homogenized cells were centrifuged at 700 g to eliminate iron beads and cell remnants. The homogenates were then ultracentrifuged at 40,000 g for 30 minutes, and finally the pellet was resuspended and stored at -80°C until further use.

### Influx assay

The influx assay was performed as previously described [27,28]. Briefly, osteoclast membranes were incubated in reaction buffer [27]. The reaction was incubated at room temperature for 30 minutes to obtain a steady state. Then the reaction was initiated by addition of ATP at a concentration of 5 mM, and immediately after the plate was read in a plate reader using excitation 492 nm and emission 535 nm. The fluorescence was read every 15 seconds for three minutes. The results are presented as the slope of the influx curves in percent of the vehicle, which represents the rate of the acidification (ΔF/Δt).

### Immunoblotting

Total cell lysates were prepared by lysing the osteoclasts in RIPA+++ buffer for 5 min [12]. The lysates were centrifuged at 15,000 g for 30 min to remove any cell debris left. Osteoclast membranes were prepared as described in the paragraph "osteoclast microsomes". Protein concentrations were measured using the Bio-Rad DC protein measurement assay. Ten micrograms of total protein for either the lysate or the membranes were loaded onto a SDS-PAGE gel in sample buffer containing 10 mM dithiotreitol, and electroblotted onto nitrocellulose membranes. The quality of the protein loading was checked by Ponceau Red staining. The membranes were then blocked in TBS-T (50 mM Tris-base pH 7.5, 100 mM NaCl, 0.1% Tween-20) containing 5% skim milk powder for 1 h at ambient temperature. This was followed by overnight incubation at 4°C with the correct dilution of the primary antibodies against PKC (A-3) (Santa Cruz) and V-ATPase B2 (Santa Cruz). This was followed by incubation with the corresponding horseradish peroxidase-conjugated secondary antibody for 1 h at ambient temperature. Finally, the results were visualized using the ECLTM kit (Amersham Pharmacia Biotech).

### Statistical analysis

Statistical analyses were performed using one-way analysis of variance followed by Dunnett's multiple comparison tests. Bartlett's test was used to assess variance homogeneity. Statistical significance is indicated by the number of asterisks, p < 0.05*, P < 0.01** and p < 0.001***.

## Results

### Protein kinase inhibitors in osteoclasts

51 protein kinase inhibitors were tested at 10 μM and at 50 μM (data not shown, since 50 μM led to more toxicity, without any more specific effects) in a panel of osteoclastic acidification and resorption assays revealing that several inhibitors were positive in bone resorption, i.e. the c-src tyrosine kinase inhibitors PP1 and PP2 as expected (Table 1 &[30]). In addition, we found that the tyrosine kinase inhibitor Tyrphostin 47 inhibited acid influx, as previously published, on the other hand Genistein, another tyrosine kinase inhibitor, did not show any inhibitory effects in the tested assays [24]. The mTOR inhibitor Rapamycin showed a minor inhibition of acid influx and bone resorption, without affecting survival, although this was expected from earlier studies [31,32]. All in all few compounds were positive in all assays, and in these cases other inhibitors of the same target failed to reproduce the data indicating that the effects were due to non-specific effects (data not shown). However, inhibitors, which are speculated to inhibit PKC showed consistent inhibition of both acid secretion and bone resorption, and thus were analysed in detail. All the results are summarised in Table 1.

**Table 1:** Summary of data for all inhibitors

Target	Inhibitor	IC50 (M)	**Acridine orange 45 min**.	Acridine orange Quenching	Influx - % Inh. at 10 μM	Influx Quenching	Resorption - % Inh at 10 μM	Alamar - % Inh. at 10 μM
**PDGFRK**	AG-370	20	No	No	20	Yes	0	0
	AG-1296	1	No	No	15	Yes	0	0

**EGFRK/PDGFRK**	AG-494	1.2	No	No	55	No	0	0
	Tyrphostin 46	9.2	No	No	0	No	0	0

**EGFRK**	Lavendustin A	0.011	No	No	0	No	0	0
	RG-14620	3	No	No	0	No	0	0
	Tyrphostin 23	35	No	No	45	Yes	0	0
	Tyrphostin 25	3	No	No	34	Yes	0	0
	Tyrphostin 47	2.4	No	No	85	No	0	0
	Tyrphostin 51	0.8	No	No	0	No	0	0
	Tyrphostin AG1478	0.003	No	No	0	No	43	0
	Erbstatin (a)	0.77	No	No	0	No	0	0
	Erlotinib	1	No	No	0	No	100	100

**NGFRK**	AG-879	10	No	No	100	No	22	0

**EGFRK/CaMKII**	Lavendustin C	2	No	No	0	No	0	0

**CaMKII**	KN-62	0.9	No	No	0	No	63	0
	KN-93	0.37	No	No	40	No	0	

**MEK1/2**	PD-98059	2	No	No	0	No	0	0
	U-0126	0.072	No	No	0	No	0	0

**p38MAPK**	SB203580	0.07	No	No	0	No	0	0

**PKA, PKG, MLCK, PKC**	H-7	3.0	No	No	0	No	0	0
	H-9	1.9	No	No	0	No	0	0

***PKC***	*GF-109203X*	*0.02*	*Yes*	*No*	*100*	*No*	*100*	*0*
	*Hypericin*	*3.4*	*No*	*No*	*82*	*Yes*	*100*	*95*
	*Ro 31-8220*	*0.01*	*No*	*No*	*79*	*Yes*	*100*	*100*
	*Sphingosine*	*1-3*	*No*	*No*	15*	*No*	*0*	*0*
	*Palmitoyl-DL-*	*25*	*No*	*No*	17*	*No*	*0*	*0*
	*Carnitine-Cl*							

***PKCδ***	*Rottlerin*	*3-6*	*Yes*	*No*	*100*	*No*	*100*	*90*

***PKCα/γ***	*HBDDE*	*43*	*No*	*No*	*58*	*Yes*	*0*	*0*

**PKA**	H-89	0.048	No	No	0	No	0	0

**PKA/PKG**	H-8	0.48	No	No	0	No	0	0
	HA-1004	30	No	No	0	No	0	0
	HA-1077	1.6	No	No	0	No	0	0

**HER1-2**	AG-825	0.35	No	No	43	Yes	0	0

**Tyrosine**	Tyrphostin AG1288	21	No	No	88	No	0	0
**Kinases**	Tyrphostin AG1295	25	No	No	0	No	0	0
	Genistein	0.002	No	No	0	No	0	0

**IRK**	HNMPA	10	No	No	0	No	0	0

**JNK**	SP 600125	0.04	No	No	0	No	0	0

**p56 lck**	Damnacanthal	0.017	No	No	0	No	47	0

**Syk**	Piceatannol	10	No	No	0	No	0	0

**Src family**	PP1	0.005	No	No	0	No	78	0
	PP2	0.004	No	No	0	No	100	45

**JAK-2**	AG-490	~5	No	No	0	No	0	0

**ERK2, CK1, CK2**	5-Iodotubercidin	0.4	No	No	0	No	100	100

**cRAF**	GW 5074	0.009	No	No	80	Yes	58	0

**IKK pathway**	BAY 11-7082	5	No	No	100	No	0	0

**GSK-3β/CDK5**	Indirubin-3'-monoxime	0.022	No	No	0	No	72	0

**mTOR**	Rapamycin	0.010	No	No	46	No	40	0

**Neg. Cont. Tyr Kinase Inhibitor**	Tyrphostin 1	NA	No	No	0	No	0	0

**Neg. Cont. Genistein**	Diadzein	NA	No	No	0	No	0	0

The table summarizes data for the acidification assay using acridine orange (45 minute incubation) and the %-inhibition in the acid influx assay at 10 μM. It is stated whether the compounds quench the acridine orange signal. The %-inhibition in the bone resorption and AlamarBlue assay are also provided for each inhibitor tested. *Indicates auto fluorescence of the compound. Text in italics shows the compounds shown in the figures. # indicates lowest IC50 values obtained in relevant assays http://www.enzolifesciences.com. NA indicates not applicable (for negative controls).

### The effect of potential PKC inhibitors on lysosomal acidification in mature human osteoclasts

**Figure 1:**
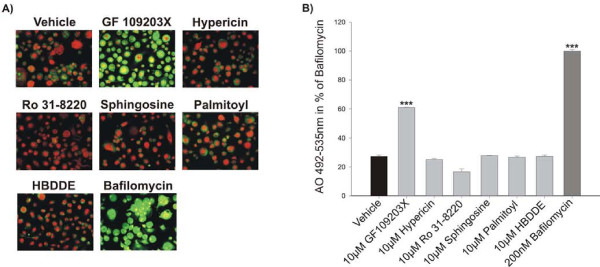
**The effect of potential PKC inhibitors on acid secretion**. Human CD14+ monocytes were isolated from blood and seeded in 96 well plates and cultured in the presence of 25 ng/ml M-CSF and RANKL for 12 days. The mature human osteoclasts were incubated for 45 minutes with acridine orange alone or in combination with the following compounds: GF109203X, Hypericin, Ro31-8220, Sphingosine, Palmitoyl-DL-carnitine-Cl, HBDDE or Bafilomycin A1 (200 nM) as a positive control. All the results are presented as % of Bafilomycin. A) Acridine Orange pictures showing the effect of the inhibitors. B) Acridine Orange readings of the green wavelength (Excitation 492 nm - emission 535 nm) was measured using a SpectraMax M5 showing the effect of the inhibitors. The results are representative of three individual experiments performed in quadruplicates. The asterisks indicate significant differences between vehicle and inhibitor treated conditions.

**Figure 2:**
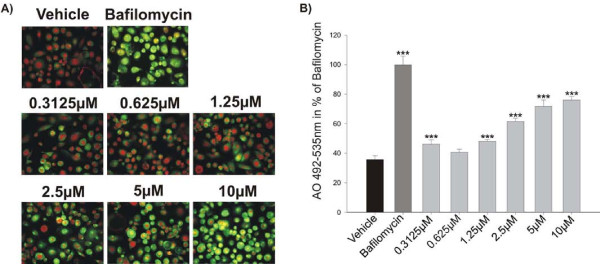
**Effect of Rottlerin on acid secretion**. Human CD14+ monocytes were isolated from blood and seeded in 96 well plates and cultured in the presence of 25 ng/ml M-CSF and RANKL for 12 days. The mature human osteoclasts were incubated for 45 minutes with acridine orange alone or in combination with different concentrations of Rottlerin or Bafilomycin A1 (200 nM) as a positive control. All the results are presented as % of Bafilomycin. A) Acridine Orange pictures showing the effect of Rottlerin. B) Acridine Orange readings of the green wavelength (Excitation 492 nm - emission 535 nm) was measured using a SpectraMax M5 showing the effect of Rottlerin. The results are representative of three individual experiments performed in quadruplicates. The asterisks indicate significant differences between vehicle and inhibitor treated conditions.

The acridine orange assay is based on a dye that fluoresces bright orange at lysosomal pH, and becomes green at neutral pH. It has previously been shown to be useful in relation to quantification of lysosomal pH changes in whole cells, as well as microsomal membranes [27,33], and therefore we used it to assess the effects of various potential PKC inhibitors in detail. GF109203X, Hypericin, Ro31-8220, Sphingosine, HBDDE and Palmitoyl-DL-carnitine-Cl were analyzed, and of these inhibitors, only GF109203X showed inhibition of lysosomal acidification and only at the 45 minute time point (Figure 1A), whereas the others were ineffective. These data were confirmed using quantitative analysis (Figure 1B). All inhibitors were tested after 45 minutes, 4 and 24 hours. However, only the 45 minutes results are shown in figure 1. In addition, Rottlerin was characterized in detail. Rottlerin inhibited lysosomal acidification already after 45 minutes incubation both in the qualitative and the quantitative assay. Due to the potent inhibition observed using Rottlerin further concentrations were tested, and as seen in figure 2, these data clearly show that Rottlerin dose-dependently inhibits lysosomal acidification at 45 minutes (Figure 2), 4 and 24 hours (data not shown). In the acidification experiments Bafilomycin A1 was used as a positive control in accordance to previously published studies [27,33].

### The effect of potential PKC inhibitors on acid influx in human osteoclast microsomes

**Figure 3:**
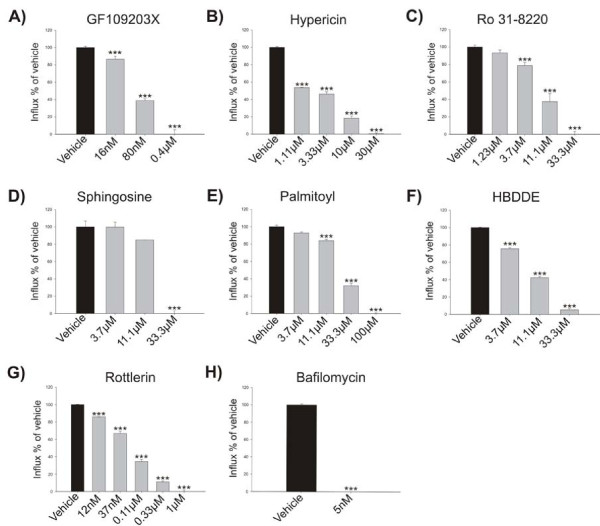
**The effect of potential PKC inhibitors on acid influx**. Acid influx in microsomes isolated from human mature osteoclasts was investigated using the dye acridine orange as described in the materials and methods section. All compounds were tested in a dose-response. A) GF109203X, B) Hypericin, C) Ro31-8220, D) Sphingosine, E) Palmitoyl-DL-carnitine-Cl, F) HBDDE, G) Rottlerin or H) Bafilomycin A1. The results are representative of three individual experiments performed in quadruplicates. The asterisks indicate significant differences between vehicle and inhibitor treated conditions.

To further characterize the effects of the inhibitors, we used a membrane-based acid influx assay based on microsomes previously shown to be enriched in ClC-7 indicating a high content of lysosomes, which are the desired sub-cellular fraction [27,28]. This assay is based on microsomes from human osteoclasts and it is highly sensitive to the V-ATPase inhibitor Bafilomycin A1, which was used as a positive control (Figure 3H) [27,28].

GF109203X (Figure 3A), Hypericin (Figure 3B), and Ro31-8220 (Figure 3C) inhibited acid influx albeit with different potencies, whereas the compounds Sphingosine (Figure 3D) and Palmitoyl-DL-carnitine Cl (Figure 3E) showed only low levels of inhibition, likely due to low potency, or alternatively due to phase partitioning into the lipid bilayer since these molecules are lipid-like. Of these general PKC inhibitors, GF109203X inhibited acid influx potently. In addition, Rottlerin inhibited the acid influx potently and to the same level as GF109203X (Figure 3G), while HBDDE showed some inhibition of acid influx (Figure 3F).

### The effect of potential PKC inhibitors on bone resorption by human osteoclasts

**Figure 4:**
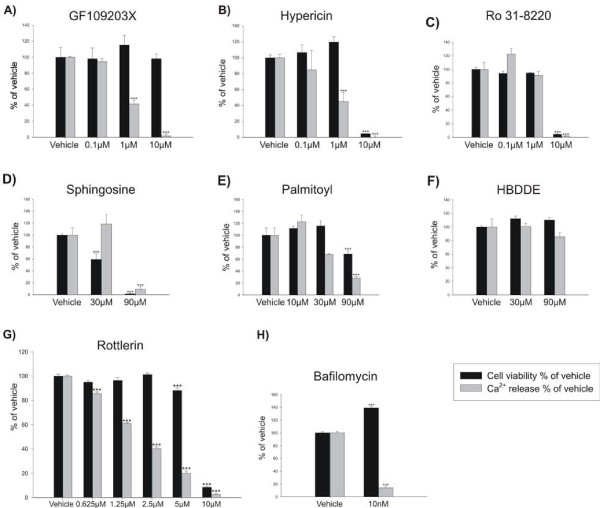
**The effect of potential PKC inhibitors on osteoclastic bone resorption**. Mature human osteoclasts were seeded on cortical bovine bone slices, and allowed to attach for 3 hours. Hereafter, dose-responses of the following compounds: A) GF109203X, B) Hypericin, C) Ro31-8220, D) Sphingosine, E) Palmitoyl-DL-carnitine-Cl, F) HBDDE, or G) Rottlerin was added to the cells. H) Bafilomycin A1 was used as a positive control. After five days of culture calcium release was measured in the culture supernatants. AlamarBlue was used to measure the cell viability after five days of culture. The results are presented as relative inhibition compared to vehicle treated osteoclasts. The results are representative of three individual experiments performed in quadruplicates. The asterisks indicate significant differences between vehicle and inhibitor treated conditions.

To investigate whether the effects of the inhibitors in the acidification assays were paralleled by inhibition of bone resorption by human osteoclasts, the different compounds were tested in a dose-response, again using Bafilomycin A1 as a positive control (figure 4H) [27,28].

All the inhibitors, except HBDDE, reduced bone resorption (Figure 4 and table 1), and their potencies in the resorption assay correlated well with the potencies observed in the acidification-based assays, with GF109203X being the most potent and Palmitoyl-DL-Carnitine Cl the least potent. Some toxicity of the compounds was observed. Only GF109203X was not toxic at the tested concentrations up to 10 μM (figure 4A), while Hypericin (figure 4B) showed toxicity at 10 μM but not at 1 μM and therefore a separation between inhibition of resorption and reduction of cell viability was seen. Palmitoyl-DL-Carnitine D1 (figure 4E) inhibited bone resorption at a high concentration, and at 90 μM the compound showed toxicity, thus making it difficult to distinguish real anti-resorptive effects from toxicity. In addition, Ro31-8220 and Sphingosine exhibited toxic effects (Figure 4C and 4D).

Rottlerin potently inhibited bone resorption (Figure 4G), whereas HBDDE had no effect (Figure 4F). Furthermore, Rottlerin reduced cell viability; however, as seen for the other inhibitors there was a clear distinction between the effect on bone resorption and the effect on cell viability.

### Detection of PKC by Western blotting

**Figure 5:**
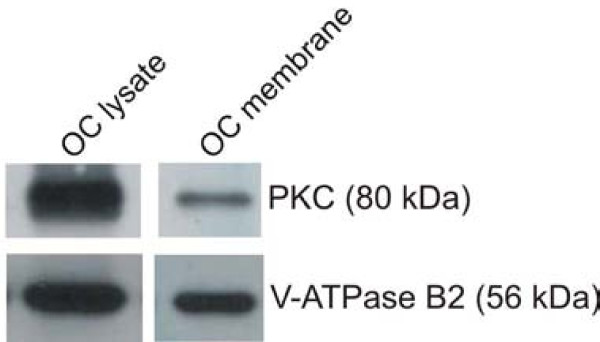
**Detection of PKC in both osteoclast membranes and osteoclast lysate**. CD14+ monocytes were cultured in the presence of M-CSF and RANKL for 12 days to mature osteoclasts were present. The osteoclasts were either used to make a lysate of mature human osteoclasts using RIPA+++, or they were used for microsome isolation. The expression of PKC, together with V-ATPase B2 as a positive control, was analyzed by loading 10 μg of lysates and microsomes on SDS-PAGE gels with subsequent immunoblotting.

To ensure that PKC was present in the microsomes, isolated from the human osteoclasts, used to analyze acid influx, Western blotting was performed. As a reference a whole cell lysate from human osteoclasts was also analyzed. PKC (80 kDa) was found in both the osteoclast membranes and in the osteoclast lysate (Figure 5). In addition, V-ATPase B2 was used as a positive control and was shown to be expressed in both osteoclast lysate and osteoclast membranes as expected [28,34].

## Discussion

Previous studies have indicated that various types of protein kinases are involved in acid production by osteoclasts from various species; however, whether this is true for pure human osteoclasts was not clear. We have used a panel of inhibitors targeting a broad range of protein kinases in a recently published series of assays [27] to investigate how acid secretion and bone resorption by mature human osteoclasts are controlled.

We found that very few of the inhibitors inhibited more than one process, if any at all, in the osteoclasts (see table 1), although the inhibitors were used at concentrations, which often far exceeded their reported IC50 values. Surprisingly our data showed that the c-src kinase inhibitors PP1 and PP2 had no effect on acidification, although this has previously been published using avian osteoclasts [26]. As expected both c-src inhibitors reduced bone resorption [30]. One possible explanation for this discrepancy is the species difference, as previous studies have indicated that the regulation of acid secretion between human and avian osteoclasts is different also with respect to the chloride channels involved [11,26-28]. Further supporting the difference between human and avian osteoclasts, we did not find any inhibitory effects of Genistein, neither on resorption nor acid secretion, which is in contrast to the findings of Williams et al. [24]. Furthermore, other studies have highlighted that Genistein reduces bone resorption [35-37], but these results were found in differentiating osteoclasts. In addition, Genistein has been indicated to activate PPARγ [38], a receptor involved in osteoclastogenesis, again showing that it attenuates osteoclastogenesis [39]. With respect to effects of Genistein, and the other general tyrosine kinase inhibitors (Tyrphostin AG1288 and Tyrphostin AG1295) on mature osteoclasts, we were surprised that they did not have any effects on bone resorption or osteoclast viability, since these effects were expected due to inhibition of c-src and c-fms [24,40-43]. We speculate that the combination of short time span, the low potency and selectivity of these compounds combined with the high doses of RANKL and M-CSF used in the culture system are the causes for the lack of effect of these compounds.

In osteoclasts of various origin both PKA and PKC have been associated with acid secretion under different circumstances [23,25], and thus their roles in the regulation of acid secretion were of high interest; however, no effects of the PKA inhibitors were detected. This correlates with the findings of Kajiya et al. [23], who found that PKA activators inhibited acid secretion in rat osteoclasts, and that PKA inhibitors protected against calcitonin mediated inhibition of acid secretion. In contrast, it was shown that inhibition of PKA-cAMP signaling reduced bone resorption by mouse osteoclasts [44], however, we could not reproduce these findings using PKA inhibitors in the human system, indicating that different species of osteoclasts utilize different signaling cascades to control bone resorption.

The most potent inhibitors of acid influx and bone resorption were all compounds indicated to inhibit PKC. The most potent inhibitor of acid influx and bone resorption was GF109203X, which is known to be somewhat selective for PKC [45]. In the cell-based acidification assay GF109203X inhibited only at 45 minutes, but not at the other time points, due to a yet unidentified reason. Interestingly Rottlerin, a molecule indicated to inhibit the PKCδ isoform was equally potent as GF109203X but in addition it also inhibits the acidification in intact human osteoclasts. These data indicate that PKC and maybe more specific PKCδ plays a role in controlling acid secretion, and thus bone resorption by human osteoclasts. These data correlate well with a study implicating PKC as involved in the acid secretion in avian osteoclasts [25]. Furthermore, studies have indicated that PKC is involved in transient shape changes in osteoclasts [46,47]; however, whether these changes have anything to do with lowered acidification remains to be clarified.

Toxicity was observed for Rottlerin in the bone resorption assay, but only at the 10 μM concentration. However, the inhibitor effect of Rottlerin on acid influx and bone resorption was apparent at lower doses, indicating that inhibition of acid influx and bone resorption is through PKC inhibition at low concentrations, while the high concentrations non-specific effects lead to toxicity, such as those described in the following section.

However, although Rottlerin has been shown to be a potent inhibitor of PKCδ [48], others have shown that Rottlerin has several other effects, and for example it strongly suppresses CHK2, PLK1, PIM3, SRPK1 [49], p38-MAPK, PKA and GSK-3b [50]. Furthermore, it has been shown that Rottlerin decreases RANK expression in macrophages, most likely by a PKC-independent pathway [51]. However, the cells used by Kang et al. [51] were U937 cells, which are used to study differentiation of monocytes to macrophages. An osteoclasts precursor cell cannot be compared to the system with mature human osteoclasts used in this study. In addition, the RANK expression does not affect the acidification, and Rottlerin seems to inhibit acidification in the mature human osteoclasts.

Like Rottlerin, GF109203X has also been shown to have other effects than as a PKC inhibitor. It has for example been shown to inhibit C1q-induced P-selectin expression [52], inhibition of activated ERK [53], and inhibition of NHE1 activity [54].

These findings indicate that both Rottlerin and GF109203X are too weak and non-specific inhibitors to be useful in cell-based studies. Furthermore, other off target effects of Rottlerin on mitochondrial function [55,56], and as a protonophore [57], question whether the effect we observed is indeed through inhibition of PKCδ. A protonophore will collapse all acid transport [58], however, it appears unlikely that collapsing all proton gradients will not affect osteoclast survival, and thus we speculate that the inhibition of resorption is unrelated to the protonophore effect. Furthermore, other inhibitors indicated to inhibit PKC also reduced acid secretion and bone resorption, potentially indicating a role of PKC in osteoclast-mediated acidification, although the specificity of all inhibitors should be interpreted with skepticism [50,59].

With respect to the commercially reported *in vitro *IC50 values, our data do not always correlate well with these, as underlined by the fact that both rottlerin and GF109203X both are very potent in our assays, and yet their *in vitro *IC50 values are far apart (see table 1). Furthermore, as illustrated throughout the manuscript IC50 values are highly assay dependent, and thus comparison of IC50 values between assays is difficult and should be done considering all the factors in play, such as membrane-permeability, access to the ruffled border, and the assay itself.

Furthermore, the discrepancies between acid influx and acidification in intact osteoclasts are not fully clear yet. Some of the inhibitors are effective inhibitors of bone resorption and acid influx, but they do not inhibit the acidification in whole cells. This can be because of the concentration and time-line used for the acidification study in whole cells. The bone resorption assay is a 5-day assay and could lead to more false positives due to this, compared to the acidification assay in which up to 24 hours incubation were tested. Henriksen et al. [27] have previously shown that the high concentrations needed to observe inhibition in the cell-based acridine orange assay can lead to unclear results. In addition, the acid influx data often correlates better with the effects on bone resorption. These findings are further illustrated by the discrepancies between the time-line for inhibition of cell-based acridine orange between GF109203X and Rottlerin. However, in acid influx assay problems due to quenching of the acridine orange signal are seen leading to false positive in the assay. Confirming the relevance of studying PKC in membrane fractions, we found that PKC is indeed present in the osteoclast microsomes, and since it is well-known that PKC can be found in two conformations; an inactive and an active form, of which the active is membrane-bound [60-62]. Thus, the system used in the influx assay contains PKC in its active membrane bound conformation.

For the validation of the results found in this study, using siRNA would be of interest, and could in the future provide important data. However, due to difficulties in getting robust transfection and knock-down in human osteoclasts, this has not yet been feasible.

## Conclusions

In this study we presented the analysis of a panel of protein kinase inhibitors in acidification of the resorption lacunae and bone resorption by human osteoclasts. However, it should be noted that some of the results are clouded by the difficulties involved in separating toxic effects from relevant inhibitory effects, as well as separating inhibition of fluorescent signals from quenching related effects, especially at the high concentrations used for some of the compounds. Furthermore, the specificity of the inhibitors is often not very high, and this is clearly illustrated by the fact that one of the most potent inhibitor of bone resorption and acid secretion, Rottlerin, has been indicated to exert a function as a proton-ionophore [63], which thus would explain its highly potent effect in all the assays. However, the compound is not overtly toxic in the long term cultures used for testing bone resorption, which is surprising for a compound eliminating all proton gradients in a whole cell. Furthermore, our finding that both GF109203X and Rottlerin inhibit acid secretion and bone resorption potently, support a role for PKC in the acidification process in human osteoclasts.

## Competing interests

Morten A. Karsdal is currently employed by and owns stocks in Nordic Bioscience. Mette G. Sørensen and Kim Henriksen are currently employed by Nordic Bioscience but own no stocks in the company. Jean A Boutin and Olivier Nosjean are currently employed by Institut de Recherche Servier (IdRS). All other authors have no conflicts of interest.

## Authors' contributions

MGS designed the experiments, performed the acridine orange analyses, the influx experiments, the bone resorption experiments, and drafted the manuscript. MAK and KH participated in experiment design and have helped to draft the manuscript. MHD was responsible for collection of the human blood samples. JAB and ON helped to draft the manuscript. All authors have read and approved the final manuscript.

## Pre-publication history

The pre-publication history for this paper can be accessed here:

http://www.biomedcentral.com/1471-2474/11/250/prepub

## References

[B1] Baron R General Principles of Bone Biology In Primer on the Metabolic Bone Diseases and Disorders of Mineral Metabolism 2005 1 8

[B2] Marks SC Hermey DC The Structure and Development of Bone Principles of Bone Biology 1996 California, USA.: Academic press 3 15

[B3] Karsdal MA Martin TJ Bollerslev J Christiansen C Henriksen K Are nonresorbing osteoclasts sources of bone anabolic activity? J Bone Miner Res 2007 22 487 494 10.1359/jbmr.070109 17227224

[B4] Seeman E Delmas PD Bone quality--the material and structural basis of bone strength and fragility N Engl J Med 2006 354 2250 2261 10.1056/NEJMra053077 16723616

[B5] Sundquist K Lakkakorpi P Wallmark B Vaananen K Inhibition of osteoclast proton transport by bafilomycin A1 abolishes bone resorption Biochem Biophys Res Commun 1990 168 309 313 10.1016/0006-291X(90)91709-2 2139331

[B6] Taranta A Migliaccio S Recchia I Caniglia M Luciani M De Rossi G Genotype-phenotype relationship in human ATP6i-dependent autosomal recessive osteopetrosis Am J Pathol 2003 162 57 68 12507890 10.1016/S0002-9440(10)63798-4 PMC1851135

[B7] Baron R Neff L Louvard D Courtoy PJ Cell-mediated extracellular acidification and bone resorption: evidence for a low pH in resorbing lacunae and localization of a 100-kD lysosomal membrane protein at the osteoclast ruffled border J Cell Biol 1985 101 2210 2222 10.1083/jcb.101.6.2210 3905822 PMC2114017

[B8] Li YP Chen W Liang Y Li E Stashenko P Atp6i-deficient mice exhibit severe osteopetrosis due to loss of osteoclast-mediated extracellular acidification Nat Genet 1999 23 447 451 10.1038/70563 10581033

[B9] al Awqati Q Chloride channels of intracellular organelles Curr Opin Cell Biol 1995 7 504 508 10.1016/0955-0674(95)80006-9 7495569

[B10] Graves AR Curran PK Smith CL Mindell JA The Cl(-)/H(+) antiporter ClC-7 is the primary chloride permeation pathway in lysosomes Nature 2008 18449189 10.1038/nature06907

[B11] Schlesinger PH Blair HC Teitelbaum SL Edwards JC Characterization of the osteoclast ruffled border chloride channel and its role in bone resorption J Biol Chem 1997 272 18636 18643 10.1074/jbc.272.30.18636 9228032

[B12] Henriksen K Gram J Schaller S Dahl BH Dziegiel MH Bollerslev J Characterization of osteoclasts from patients harboring a G215R mutation in ClC-7 causing autosomal dominant osteopetrosis type II Am J Pathol 2004 164 1537 1545 15111300 10.1016/S0002-9440(10)63712-1 PMC1615650

[B13] Kornak U Kasper D Bosl MR Kaiser E Schweizer M Schulz A Loss of the ClC-7 chloride channel leads to osteopetrosis in mice and man Cell 2001 104 205 215 10.1016/S0092-8674(01)00206-9 11207362

[B14] Gelb BD Shi GP Chapman HA Desnick RJ Pycnodysostosis, a lysosomal disease caused by cathepsin K deficiency Science 1996 273 1236 1238 10.1126/science.273.5279.1236 8703060

[B15] Saftig P Hunziker E Wehmeyer O Jones S Boyde A Rommerskirch W Impaired osteoclastic bone resorption leads to osteopetrosis in cathepsin-K-deficient mice Proc Natl Acad Sci USA 1998 95 13453 13458 10.1073/pnas.95.23.13453 9811821 PMC24840

[B16] Frattini A Orchard PJ Sobacchi C Giliani S Abinun M Mattsson JP Defects in TCIRG1 subunit of the vacuolar proton pump are responsible for a subset of human autosomal recessive osteopetrosis Nat Genet 2000 25 343 346 10.1038/77131 10888887

[B17] Kornak U Schulz A Friedrich W Uhlhaas S Kremens B Voit T Mutations in the a3 subunit of the vacuolar H(+)-ATPase cause infantile malignant osteopetrosis Hum Mol Genet 2000 9 2059 2063 10.1093/hmg/9.13.2059 10942435

[B18] Cleiren E Benichou O Van Hul E Gram J Bollerslev J Singer FR Albers-Schonberg disease (autosomal dominant osteopetrosis, type II) results from mutations in the ClCN7 chloride channel gene Hum Mol Genet 2001 10 2861 2867 10.1093/hmg/10.25.2861 11741829

[B19] Schaller S Henriksen K Sveigaard C Heegaard AM Helix N Stahlhut M The chloride channel inhibitor n53736 prevents bone resorption in ovariectomized rats without changing bone formation J Bone Miner Res 2004 19 1144 1153 10.1359/JBMR.040302 15176998

[B20] Karsdal MA Henriksen K Sorensen MG Gram J Schaller S Dziegiel MH Acidification of the osteoclastic resorption compartment provides insight into the coupling of bone formation to bone resorption Am J Pathol 2005 166 467 476 15681830 10.1016/S0002-9440(10)62269-9 PMC1602325

[B21] Teti A Colucci S Grano M Argentino L Zambonin ZA Protein kinase C affects microfilaments, bone resorption, and [Ca2+]o sensing in cultured osteoclasts Am J Physiol 1992 263 C130 C139 1636672 10.1152/ajpcell.1992.263.1.C130

[B22] Beene DL Scott JD A-kinase anchoring proteins take shape Curr Opin Cell Biol 2007 19 192 198 10.1016/j.ceb.2007.02.011 17317140 PMC3521038

[B23] Kajiya H Okamoto F Fukushima H Okabe K Calcitonin inhibits proton extrusion in resorbing rat osteoclasts via protein kinase A Pflugers Arch 2003 445 651 658 12632184 10.1007/s00424-002-0989-4

[B24] Williams JP Jordan SE Barnes S Blair HC Tyrosine kinase inhibitor effects on avian osteoclastic acid transport Am J Clin Nutr 1998 68 1369S 1374S 9848501 10.1093/ajcn/68.6.1369S

[B25] Williams JP Thames AM McKenna MA McDonald JM Differential effects of calmodulin and protein kinase C antagonists on bone resorption and acid transport activity Calcif Tissue Int 2003 73 290 296 10.1007/s00223-002-0012-2 14667143

[B26] Edwards JC Cohen C Xu W Schlesinger PH c-Src control of chloride channel support for osteoclast HCl transport and bone resorption J Biol Chem 2006 281 28011 28022 10.1074/jbc.M605865200 16831863 PMC1808340

[B27] Henriksen K Sorensen MG Jensen VK Dziegiel MH Nosjean O Karsdal MA Ion transporters involved in acidification of the resorption lacuna in osteoclasts Calcif Tissue Int 2008 83 230 242 10.1007/s00223-008-9168-8 18787885

[B28] Henriksen K Gram J Neutzsky-Wulff AV Jensen VK Dziegiel MH Bollerslev J Characterization of acid flux in osteoclasts from patients harboring a G215R mutation in ClC-7 Biochem Biophys Res Commun 2009 804 809 10.1016/j.bbrc.2008.11.145 19070589

[B29] Karsdal MA Hjorth P Henriksen K Kirkegaard T Nielsen KL Lou H Transforming growth factor-beta controls human osteoclastogenesis through the p38 MAPK and regulation of RANK expression J Biol Chem 2003 278 44975 44987 10.1074/jbc.M303905200 12933809

[B30] Furuyama N Fujisawa Y Regulation of collagenolytic protease secretion through c-Src in osteoclasts Biochem Biophys Res Commun 2000 272 116 124 10.1006/bbrc.2000.2698 10872813

[B31] Glantschnig H Fisher JE Wesolowski G Rodan GA Reszka AA M-CSF, TNFalpha and RANK ligand promote osteoclast survival by signaling through mTOR/S6 kinase Cell Death Differ 2003 10 1165 1177 10.1038/sj.cdd.4401285 14502240

[B32] Ory B Moriceau G Redini F Heymann D mTOR inhibitors (rapamycin and its derivatives) and nitrogen containing bisphosphonates: bi-functional compounds for the treatment of bone tumours Curr Med Chem 2007 14 1381 1387 10.2174/092986707780831159 17584050

[B33] Sorensen MG Henriksen K Neutzsky-Wulff AV Dziegiel MH Karsdal MA Diphyllin, a Novel and Naturally Potent V-ATPase Inhibitor, Abrogates Acidification of the Osteoclastic Resorption Lacunae and Bone Resorption J Bone Miner Res 2007 22 1640 1648 10.1359/jbmr.070613 17576165

[B34] Lee BS Holliday LS Ojikutu B Krits I Gluck SL Osteoclasts express the B2 isoform of vacuolar H(+)-ATPase intracellularly and on their plasma membranes Am J Physiol 1996 270 C382 C388 8772466 10.1152/ajpcell.1996.270.1.C382

[B35] Blair HC Jordan SE Peterson TG Barnes S Variable effects of tyrosine kinase inhibitors on avian osteoclastic activity and reduction of bone loss in ovariectomized rats J Cell Biochem 1996 61 629 637 10.1002/(SICI)1097-4644(19960616)61:4<629::AID-JCB17>3.0.CO;2-A 8806087

[B36] Yamaguchi M Gao YH Inhibitory effect of genistein on bone resorption in tissue culture Biochem Pharmacol 1998 55 71 76 10.1016/S0006-2952(97)00402-4 9413932

[B37] Li B Yu S Genistein prevents bone resorption diseases by inhibiting bone resorption and stimulating bone formation Biol Pharm Bull 2003 26 780 786 10.1248/bpb.26.780 12808286

[B38] Dang ZC Audinot V Papapoulos SE Boutin JA Lowik CW Peroxisome proliferator-activated receptor gamma (PPARgamma) as a molecular target for the soy phytoestrogen genistein J Biol Chem 2003 278 962 967 10.1074/jbc.M209483200 12421816

[B39] Wan Y Chong LW Evans RM PPAR-gamma regulates osteoclastogenesis in mice Nat Med 2007 13 1496 1503 10.1038/nm1672 18059282

[B40] El HD Gallet M Mentaverri R Sevenet N Brazier M Kamel S Imatinib mesylate (Gleevec) enhances mature osteoclast apoptosis and suppresses osteoclast bone resorbing activity Eur J Pharmacol 2006 551 27 33 10.1016/j.ejphar.2006.09.007 17049513

[B41] Ohno H Kubo K Murooka H Kobayashi Y Nishitoba T Shibuya M A c-fms tyrosine kinase inhibitor, Ki20227, suppresses osteoclast differentiation and osteolytic bone destruction in a bone metastasis model Mol Cancer Ther 2006 5 2634 2643 10.1158/1535-7163.MCT-05-0313 17121910

[B42] Recchia I Rucci N Funari A Migliaccio S Taranta A Longo M Reduction of c-Src activity by substituted 5,7-diphenyl-pyrrolo[2,3-d]-pyrimidines induces osteoclast apoptosis in vivo and in vitro. Involvement of ERK1/2 pathway Bone 2004 34 65 79 10.1016/j.bone.2003.06.004 14751564

[B43] Soriano P Montgomery C Geske R Bradley A Targeted disruption of the c-src proto-oncogene leads to osteopetrosis in mice Cell 1991 64 693 702 10.1016/0092-8674(91)90499-O 1997203

[B44] Park YG Kim YH Kang SK Kim CH cAMP-PKA signaling pathway regulates bone resorption mediated by processing of cathepsin K in cultured mouse osteoclasts Int Immunopharmacol 2006 6 947 956 10.1016/j.intimp.2006.01.005 16644480

[B45] Goekjian PG Jirousek MR Protein kinase C in the treatment of disease: signal transduction pathways, inhibitors, and agents in development Curr Med Chem 1999 6 877 903 10495357

[B46] Holloway WR Collier FM Herbst RE Hodge JM Nicholson GC Complex shape changes in isolated rat osteoclasts: involvement of protein kinase C in the response to calcitonin Calcif Tissue Int 1997 61 306 312 10.1007/s002239900340 9312201

[B47] Yamamoto Y Yamamoto Y Udagawa N Okumura S Mizoguchi T Take I Effects of calcitonin on the function of human osteoclast-like cells formed from CD14-positive monocytes Cell Mol Biol (Noisy -le-grand) 2006 52 25 31 17535751

[B48] Gschwendt M Muller HJ Kielbassa K Zang R Kittstein W Rincke G Rottlerin, a novel protein kinase inhibitor Biochem Biophys Res Commun 1994 199 93 98 10.1006/bbrc.1994.1199 8123051

[B49] Bain J Plater L Elliott M Shpiro N Hastie CJ McLauchlan H The selectivity of protein kinase inhibitors: a further update Biochem J 2007 408 297 315 10.1042/BJ20070797 17850214 PMC2267365

[B50] Davies SP Reddy H Caivano M Cohen P Specificity and mechanism of action of some commonly used protein kinase inhibitors Biochem J 2000 351 95 105 10.1042/0264-6021:3510095 10998351 PMC1221339

[B51] Kang HS Park EK Kim KH Park JY Choi JY Shin HI Receptor activator of nuclear factor-kappaB is induced by a rottlerin-sensitive and p38 MAP kinase-dependent pathway during monocyte differentiation Mol Cells 2004 17 438 445 15232218

[B52] Skoglund C Wettero J Tengvall P Bengtsson T C1q induces a rapid up-regulation of P-selectin and modulates collagen- and collagen-related peptide-triggered activation in human platelets Immunobiology 2010 20163886 10.1016/j.imbio.2009.11.004

[B53] Hanieh H Abe A Kondo Y Extracellular signal-regulated kinase (ERK) activation in chicken heterophils stimulated with phorbol 12-myristate 13-acetate (PMA), formyl-methionylleucyl-phenylalanine (fMLP) and lipopolysaccharide (LPS) Anim Sci J 2009 80 577 584 10.1111/j.1740-0929.2008.00622.x 20163623

[B54] Kato K Yasutake M Jia D Snabaitis AK Avkiran M Kusama Y Urotensin II activates sarcolemmal Na(+)/H(+) exchanger in adult rat ventricular myocytes J Cardiovasc Pharmacol 2010 55 191 197 10.1097/FJC.0b013e3181cf0074 20040885

[B55] Liao YF Hung YC Chang WH Tsay GJ Hour TC Hung HC The PKC delta inhibitor, rottlerin, induces apoptosis of haematopoietic cell lines through mitochondrial membrane depolarization and caspases' cascade Life Sci 2005 77 707 719 10.1016/j.lfs.2005.01.010 15922001

[B56] Ringshausen I Oelsner M Weick K Bogner C Peschel C Decker T Mechanisms of apoptosis-induction by rottlerin: therapeutic implications for B-CLL Leukemia 2006 20 514 520 10.1038/sj.leu.2404113 16437144

[B57] Soltoff SP Rottlerin: an inappropriate and ineffective inhibitor of PKCdelta Trends Pharmacol Sci 2007 28 453 458 10.1016/j.tips.2007.07.003 17692392

[B58] Blair HC Teitelbaum SL Tan HL Koziol CM Schlesinger PH Passive chloride permeability charge coupled to H(+)-ATPase of avian osteoclast ruffled membrane Am J Physiol 1991 260 C1315 C1324 1829326 10.1152/ajpcell.1991.260.6.C1315

[B59] Mathur A Vallano ML 2,2',3,3',4,4'-Hexahydroxy-1,1'-biphenyl-6,6'-dimethanol dimethyl ether (HBDDE)-induced neuronal apoptosis independent of classical protein kinase C alpha or gamma inhibition Biochem Pharmacol 2000 60 809 815 10.1016/S0006-2952(00)00398-1 10930535

[B60] Dutil EM Keranen LM Paoli-Roach AA Newton AC In vivo regulation of protein kinase C by trans-phosphorylation followed by autophosphorylation J Biol Chem 1994 269 29359 29362 7961910

[B61] Newton AC Protein kinase C: poised to signal Am J Physiol Endocrinol Metab 2010 298 E395 E402 10.1152/ajpendo.00477.2009 19934406 PMC2838521

[B62] Arciuch VG Alippe Y Carreras MC Poderoso JJ Mitochondrial kinases in cell signaling: Facts and perspectives Adv Drug Deliv Rev 2009 61 1234 1249 10.1016/j.addr.2009.04.025 19733603

[B63] Xu SZ Rottlerin induces calcium influx and protein degradation in cultured lenses independent of effects on protein kinase C delta Basic Clin Pharmacol Toxicol 2007 101 459 464 10.1111/j.1742-7843.2007.00143.x 17927688

